# Polypharmacy in Children with Medical Complexity: A Cross-Sectional Study in a Pediatric Palliative Care Center

**DOI:** 10.3390/children11070821

**Published:** 2024-07-04

**Authors:** Anna Zanin, Fernando Baratiri, Barbara Roverato, Daniele Mengato, Lisa Pivato, Irene Avagnina, Irene Maghini, Antuan Divisic, Francesca Rusalen, Caterina Agosto, Francesca Venturini, Franca Benini

**Affiliations:** 1 Palliative Care and Pain Service, Department of Women’s and Children’s Health, University of Padua, 35128 Padua, Italy; barbara.roverato@aopd.veneto.it (B.R.); ireneavagnina@gmail.com (I.A.); irene.maghini@aopd.veneto.it (I.M.); antuan.divisic@aopd.veneto.it (A.D.); francesca.rusalen@aopd.veneto.it (F.R.); caterina.agosto@aopd.veneto.it (C.A.); franca.benini@aopd.veneto.it (F.B.); 2 Department of Women’s and Children’s Health, University of Padua, 35128 Padua, Italy; fernando.baratiri@aopd.veneto.it; 3 Hospital Pharmacy Department, Padua University Hospital, 35128 Padua, Italy; daniele.mengato@aopd.veneto.it (D.M.); lisa.pivato@aopd.veneto.it (L.P.); francesca.venturini@aopd.veneto.it (F.V.)

**Keywords:** polypharmacy, off-label drug use, medication burden, pediatrics, children with medical complexity, pediatric palliative care

## Abstract

Background: Children with medical complexity (CMC) often require multiple medications, leading to polypharmacy, which seems to be linked to adverse effects, administration errors, and increased caregiver burden. This study aimed to describe the prevalence of polypharmacy, medication burden, off-label drug use, and associated costs. Methods: Conducted at the Pediatric Palliative Care Center of Padua, Italy, from August to October 2021, this cross-sectional observational study included patients up to 23 years old with at least one prescribed drug. Data were collected from medical records and caregiver interviews. Drug costs were collected from the Italian Medicine Agency. Descriptive statistical analysis was performed. For comparisons among categorical variables, the Chi-square test was used, and for those among continuous variables, the ANOVA test was used. Results: This study analyzed treatment regimens of 169 patients with a median age of 12.5 years (0.3–23). Polypharmacy was present in 52.7% of patients, and medication burden was observed in 44.4%, both varying significantly by primary diagnosis (*p* < 0.001). The median daily cost per patient was EUR 2.2 (IQR 0.9–7.1), with significant variation among subgroups. Only 34.6% of prescriptions were off-label. Conclusions: polypharmacy and medication burden are frequent among our CMC population, with some differences according to primary diagnosis.

## 1. Introduction

Children with medical complexity (CMC) are a growing pediatric patient population characterized by chronic illness conditions, fragility, comorbidities, a high risk of mortality, special health care needs, complex medication regimens (CMRs), and significant care burden on their families [1,2]. Their management is increasingly provided by Pediatric Palliative Care (PPC). PPC aims to care for patients with life-limiting or life-threatening conditions and their family in a multidimensional way. Goals of care for PPC are to guarantee symptom control, to avoid suffering, and to sustain families, aiming to achieve the best quality of life (QoL). In order to obtain appropriate symptom control, CMC are often exposed to CMRs and polypharmacy [3]

Polypharmacy is defined as the administration or use of multiple medications for the concomitant treatment of one or different medical disorders [4]. This term is commonly used to describe the use of five or more medications for adults, although for children, the threshold seems to be two or more medications [5,6,7,8,9,10,11,12]. However, several studies involving polypharmacy in CMC reported polypharmacy using the five or more medications definition [13,14,15]. In our study, we decided to adopt this definition because it seems to be more suitable due to the intrinsic characteristics of the CMC population, characterized by high symptom burden and several comorbidities. Polypharmacy is very common in the field of pediatric chronic disease, where polypharmacy is founded on clinical evidence in a specific and defined population [16]. Otherwise, scant data are reported for CMC, which represent a heterogeneous population for which evidence-based protocols and practice guidelines are often lacking [1,2].

Polypharmacy is associated with both benefits and harms [17]. Indeed, parents and caregivers of CMC must often deal with CMRs, with a high risk of administration errors and adherence issues. Furthermore, polypharmacy regimens in CMC often comprise unconventional administration routes and are associated with a high risk of drug–drug interactions [17,18]. Moreover, due to the lack of therapies specifically developed for pediatric patients, CMC seem to be often exposed to the off-label use of medications [19,20,21]. This represents a very common practice in the pediatric population, with estimates ranging from 9% to 99.5%, according to different settings and assessment criteria [22,23,24,25]. Despite the common use of this practice, off-label therapies can be harmful due to an augmented risk for adverse drug events (ADEs) compared with on-label use [26,27,28,29]. Considering the frailty of CMC, the issue of off-label drug use is critical and the investigation of the extension of the off-label practice is becoming more of a concern [30]. We have therefore conducted a monocentric, cross-sectional study in a referral center for PPC, with the aim to describe the population of CMC receiving polypharmacy, including the types of medications used, and assess the off-label incidence in this cohort of patients. The daily and annual cost of CMC’s treatment regimens were also assessed.

## 2. Materials and Methods

### 2.1. Study Design

This was a cross-sectional observational study performed between August and October 2021 in the Tertiary Care Pediatric Department of the University Hospital of Padua, Italy. CMC were followed at the Regional Pediatric Palliative Care Center.

This study was conducted in accordance with Good Clinical Practice (GCP) using the guidance documents and practices offered by the International Conference on Harmonization and the European directives 2001/20/CE and ISO 14155, and in agreement with the local regulations. The final protocol and its amendments were reviewed and approved by the local Ethical Committee (EC) with the number 197n/AO/21. Written informed consent was obtained from a parent and/or legal guardian.

### 2.2. Patients

This study involved all patients in care at the PPC center over the study period, with at least one prescribed drug treatment and aged less than 23 years.

### 2.3. Data Collection and Analysis

Data include information about the patient’s status and pharmacological therapies. Specifically, the following baseline characteristics were collected: gender, age, main diagnostic category and disease history duration, and “do not resuscitate” (DNR) orders. The care complexity of each patient was stratified using the ACCAPED (Scheda di Accertamento dei Bisogni Clinico Assistenziali Complessi in Pediatria) scale, a questionnaire validated in the Italian language, that evaluates PPC needs and, giving a global score, categorizes patients into low, medium, and high care complexity [31]. Regarding the data on medication regimens, for each patient, we collected the number of drugs prescribed; their correspondence to the Anatomical Therapeutic Chemical (ATC) classification; their dosage, frequency, and route of administration; correspondence with the indications of the Summary of Product Characteristics (SmPC); and their national manufacturer market prices. Three principal routes of administration were identified, enteral (including oral and via feeding tubes), parenteral (including intravenous, intramuscular, and subcutaneous), and other, grouping all the routes of administration that appeared fewer than 10 times (sublingual, endorectal, transcutaneous, inhalation, eye drops, ear drops, and nasal spray). All data were first collected from patients’ medical records. Drug therapy information was also confirmed by cross-checking with data from our Hospital Pharmacy Department databases.

### 2.4. Polypharmacy

We define polypharmacy as the current administration of five or more different drugs. Scheduled medications were assessed for polypharmacy definition [13,14,15,32]. Medications prescribed “as needed” were considered in this study but not included in frequency administration count. According to the recent article by Fraser et al. [13], we also report ten or more unique medications as a definition of “excessive polypharmacy”.

### 2.5. Medication Burden

Medication burden was defined as polypharmacy and at least 2 administrations during nights. No information on adverse events or drug interactions were collected.

### 2.6. Off-Label Drug Use

To assess the off-label drug use (OLDU), guidelines on the use of drugs issued by the Italian Medicines Agency (Agenzia Italiana del Farmaco, AIFA) were consulted [33]. OLDU is defined as any drug available on the market but used for indications not yet approved by regulatory agencies [33,34]. Even a route of administration or dosing regimen not covered by the SmPC identifies the drug as an OLDU. The SmPC, therefore, has been considered the reference point for the verification of off-label compliance. In addition, all uses not in SmPC but evaluated by AIFA as appropriate, in accordance with Italian Law No. 648/96, were not considered OLDU [35]. All the off-label cases (used for different indications, used in a different population, route of administration or dosing regimen not covered by the SmPC) were grouped into a single OLDU category; therefore, results relating to the OLDU are “cumulative”. A separate discussion should be made for medical products pertaining to compassionate use programs. These drugs, although not yet officially available on the market, cannot be considered as OLDU as their use falls within the indications of the SmPC.

### 2.7. Pharmaceutical Expenditure

With respect to the quantification of pharmaceutical expense, we chose to standardize costs based on the national manufacturer market price of each product. This price allows a more comparable figure to be obtained in the literature as it corresponds to the cost set by the manufacturer for a given product [36].

### 2.8. Statistical Analysis

Continuous data were reported as mean ± standard deviation, median, minimum, and maximum and were analyzed with the two-sided Student’s *t*-test. Categorical variables were reported as frequencies and percentages and were analyzed with the χ^2^-test with Fisher’s exact test when appropriate. Statistical significance was set at *p* < 0.05. Data were analyzed through IBM SPSS Statistics for Windows, Version 28.0.1.1

## 3. Results

### 3.1. Demographics

Treatment regimens of 169 patients with a median age of 12.5 years were analyzed. Table 1 reports their baseline characteristics. Almost half of the patients (41.4%) were affected primarily from neurological disorders and most patients had a congenital or perinatal onset (90.5%). A DNR order was given for 42 children (25.0%).

### 3.2. The ACCAPED Scale Score

Table 2 reports the average distribution of the ACCAPED scale score in the subpopulations analyzed and the distribution of patients by their complexity degree. There are three complexity degree categories: low-complexity needs (ACCAPED score ≤ 29), middle-complexity needs (ACCAPED score between 30 and 49), and high-complexity needs (ACCAPED score ≥ 50).

The mean ACCAPED scale score for each patient was 57 (±27). Patients with a primary neurological, genetic–metabolic, and oncological diagnosis resulted in the groups with the highest mean score. The mean ACCAPED scale score was significantly higher in patients with a DNR order or in patients with medication burden, relative to patients without these conditions.

By stratifying patients according to ACCAPED scale score, the majority of patients (84%) fell into the medium- and high-complexity-care groups. Furthermore, 88% of patients with a DNR order and 76% of patients with medication burden were in the high-care-complexity category.

### 3.3. Drugs Details

Table 3 shows the average number of drugs administered per day and as needed, their distribution during the day, and the classification and the principal route of administration.

**Figure 1:**
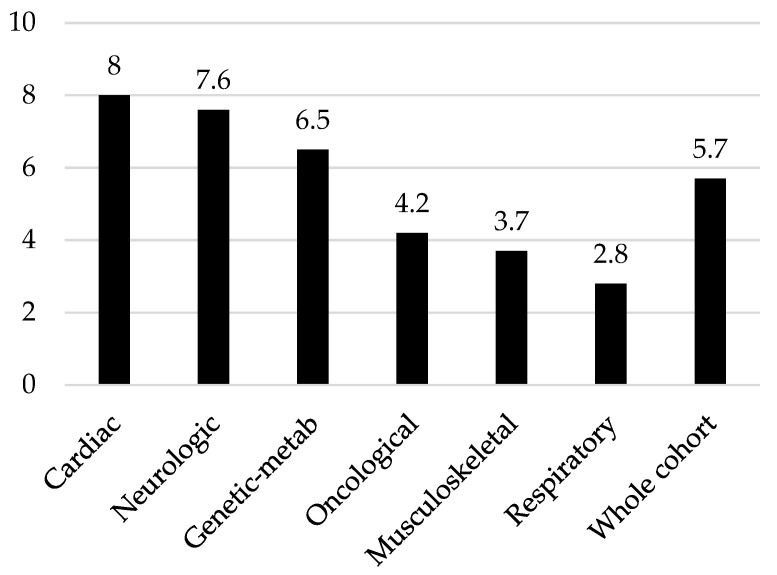
Average number of drugs administered per day.

The mean number of different drugs administered per day was 5.7 (±4.0). Patients with a primary cardiac (8.0 ± 1.4) and neurological diagnosis (7.5 ± 4.3) resulted in the groups with highest number of different drugs per day (see Figure 1).

**Figure 2:**
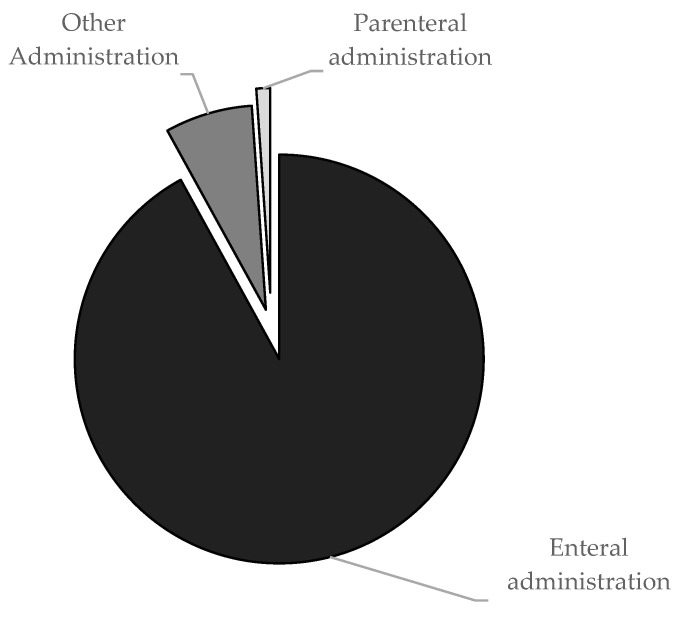
Drugs administered enterally, including oral and via enteral feeding tubes, were the most frequent (891/969, 92.0%), followed by drugs of the administration category other (67/969, 6.9%); parenteral drugs were used only in rare cases (11/969, 1.1%).

The mean number of daytime administrations (from 8:00 AM to 7:59 PM) was 5.5 (±4.4), with the highest values in patients with cardiac (10.5 ± 6.4) and neurological diagnoses (7.2 ± 4.8). The mean number of nighttime administrations (from 8:00 PM to 7:59 AM) was 2.7 (±3.0), with 4.2 (±3.3) for neurological conditions, 3.4 (±2.9) for genetic/metabolic conditions, and 3.0 (±0) for cardiac conditions. As shown in Figure 2, the most common routes of administration are the enteral, followed by the category other; the parenteral one was the least used.

**Figure 3:**
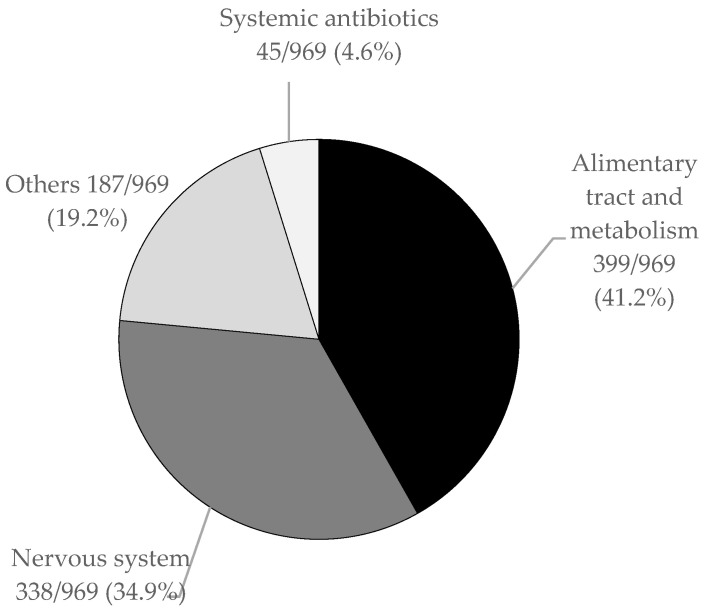
Distribution of principal drug classes according to ATC (Anatomical Therapeutic Chemical) classification.

Considering the whole cohort, a total of 969 drugs were prescribed. Each treatment regimen included a mean of 3 (±1) ATC classes. Alimentary tract and metabolism (399/969 41.2%), neurologic (338/969 34.9%), and anti-infectives for systemic use (45/969 4.6%) were the most represented drug classes (see Figure 3).

Neurologic drugs were mostly antiepileptics (53.0%). Also included within this subgroup were gabapentinoids used for the treatment of neuropathic pain, followed by psycholeptics (23.0%) and analgesics (22.0%). Details on the use of neurologic drugs are reported in Table S1 in Supplementary Materials.

### 3.4. Polypharmacy, Self-Administration, and Medication Burden

In total, 89 (52.7%) patients were on polypharmacy, 33 of whom (19.5%) were on excessive polypharmacy (see Table 1). Table 4 describes the prevalence of polypharmacy, self-administration, and medication burden. Regarding polypharmacy, it was significantly more prevalent in patients with neurological, cardiac, and genetic–metabolic primary diagnoses (*p* < 0.001), as well as in patients with a DNR order (76.2% vs. 44.9%; *p* < 0.001).

Drug self-administration was reported in 38 patients (22.5%); its prevalence was significantly different according to the primary diagnosis. It was mainly reported in patients affected by respiratory disorder (72.7%) or oncological disease (63.6%).

**Figure 4:**
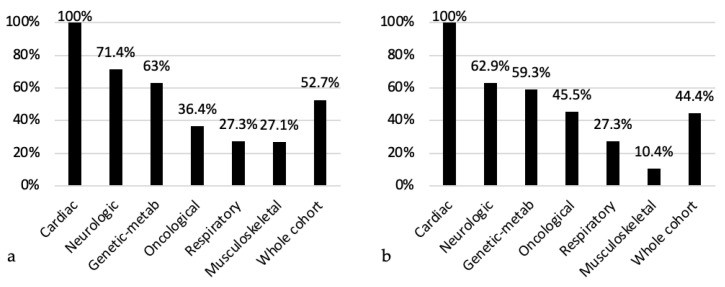
Prevalence of polypharmacy (**a**) and medication burden (**b**) according to primary diagnostic categories.

Medication burden was reported in 75 patients (44.4%). The results were significantly different according to the primary diagnosis (*p* < 0.001), with a higher prevalence in patients with neurological, cardiac, and genetic–metabolic primary diagnoses. Medication burden was more frequent among patients with a DNR order than in patients without it (73.8% vs. 35.4%; *p* < 0.001). Figure 4 shows the prevalence of polypharmacy and medication burden according to primary diagnostic categories.

### 3.5. Off-Label Drug Use

**Figure 5:**
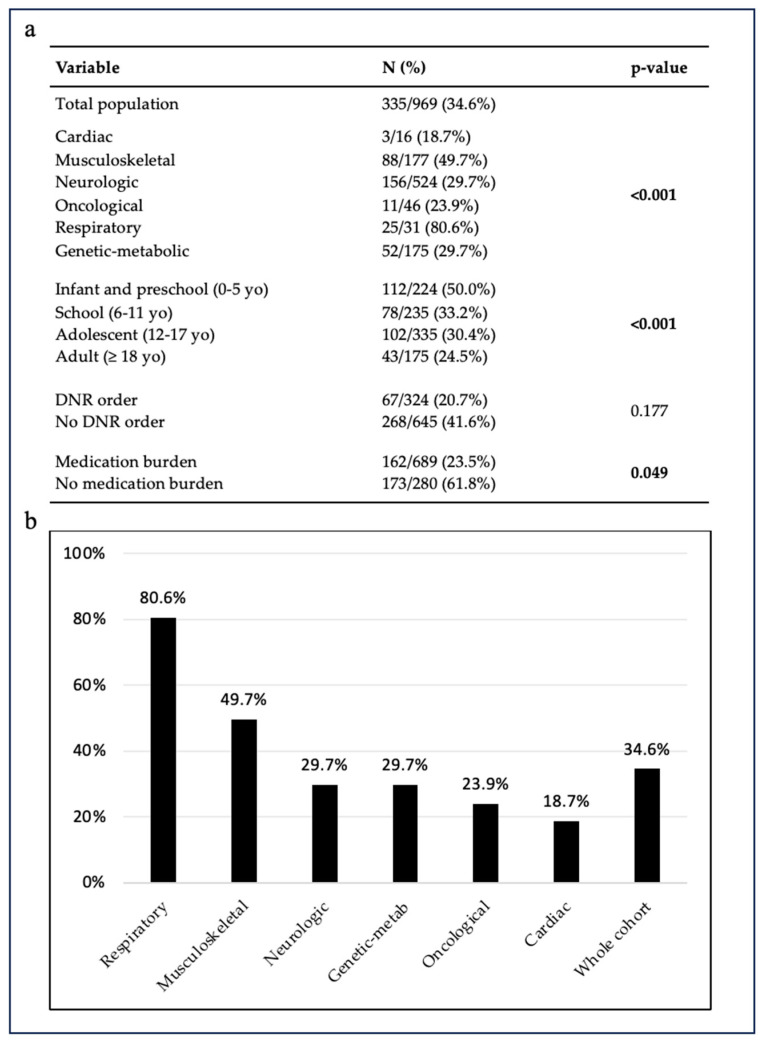
Prevalence of off–label drug use according to different subpopulations: (**a**,**b**) charted prevalence according to primary diagnostic categories.

Figure 5 shows the prevalence of OLDU in the population analyzed. Of 969 total prescriptions, 335 (34.6%) were off-label, with an increasing prevalence in younger patients such as infant and preschool children compared to adult patients (50.0% vs. 24.5%; *p* < 0.001). Comparing the off-label distribution among the diagnosis groups, respiratory (80.6%) and musculoskeletal (49.7%) showed a higher frequency of OLDU compared with other groups (*p* < 0.001). No significant difference was observed between patients with and without a DNR order. Surprisingly, the greatest use of off-label drugs was found in patients without medication burden compared to patients with it (61.8% vs. 23.5%, *p* = 0.049).

### 3.6. Pharmaceutical Expenditure

**Figure 6:**
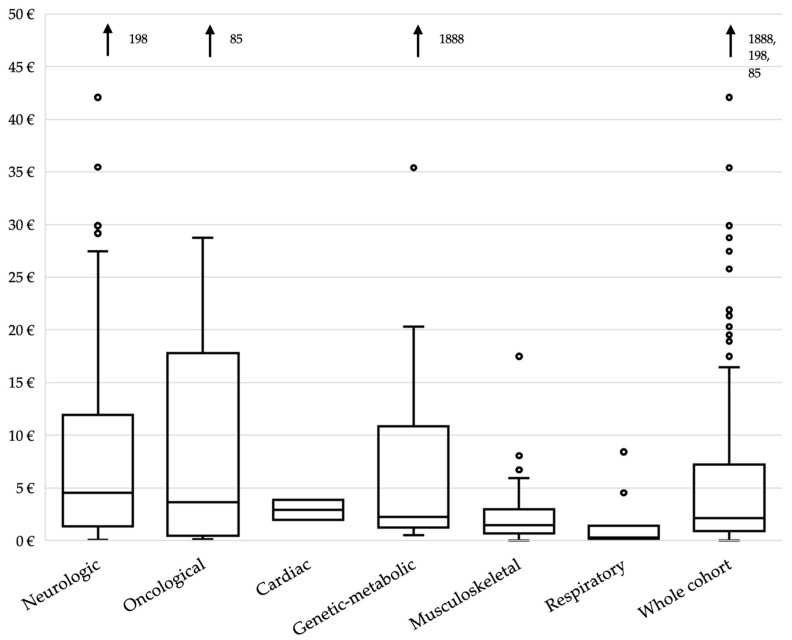
Median daily costs according to primary diagnostic categories in EUR. Black arrows indicate the value of extreme outliers which has not been included, to better show the main part of data.

Table 5 describes and compares the annual and daily cost of therapies. The median daily cost for each patient was estimated to be EUR 2.2 (IQR 0.9–7.1), with a significant variation among the subgroups going from a median daily cost for patients with a primary respiratory condition of EUR 0.3 (IQR 0.1–1.3) and EUR 4.5 (IQR 1.3–11.8) for patients with neurologic conditions, as seen in Figure 6. Median daily costs for patients with DNR orders or exposed to medication burden are significantly higher (*p* < 0.001).

## 4. Discussion

With the increased rates of diagnosed pediatric complex disease states, the prevalence of polypharmacy in the pediatric setting has increased [13,37].

The results of this study suggest that polypharmacy and medication burden are frequent among patients with medical complexities (52.7% and 44.4% of patients, respectively).

Despite some reports defining polypharmacy as with two or more medications in the pediatric population [12,38], we considered it more impactful in our population cohort to measure the complexity of medication considering polypharmacy as the administration of five or more different drugs. Also according to the recent publication of Fraser et al. [13], this last definition seems the most commonly applied and also more significant to date.

CMRs are difficult to manage for the clinicians because of the multiple possible interactions but also for caregivers that need specific training [39]. To date, there are few dedicated tools to help clinicians and pharmacists in identifying and measuring the various aspects of the burden associated with medication complexity in the adult population [40,41]. The concept of “medication-related burden” has been well described as the overall workload that is imposed on patients resulting from utilizing health care, leading to multiple negative effects, but this is not defined in pediatric patients [42,43,44]. In this study, we wanted to try to integrate polypharmacy and the caregiver’s burden using nightly administration in order to have a simple but significant and easily measurable new perspective that is more patient-oriented. In CMC, clinical and pharmacological complexities appear to be correlated. Patients with medication burden and patients with DNR orders had the highest mean score of the ACCAPED scale, 68 (±26) and 72 (±24), respectively. In this study, we used the ACCAPED scale, a new tool to evaluate clinical needs and eligibility for PPC, to stratify patients’ care complexity. However, further studies could investigate the possibility of using this scale to identify earlier the risk of medication burden for patients and their caregivers. Polypharmacy and medication burden were more frequent among patients with a primary and neurological diagnosis, with a higher incidence in patients with a DNR order. This is a possible indirect index of a higher complexity of care and more advanced state of their disease, but this aspect should be further investigated.

Of note, the most used category of drugs concerns the alimentary tract and metabolism (399 out of 969 prescribed drugs), followed by neurological drugs (338 out of 969 prescribed drugs), which are representative of symptoms that often occur across the spectrum of children suffering from conditions with a high complexity of care, despite the heterogeneity of the underlying pathologies [2]. Similarly, antibiotics are another category of drugs widely used across the underlying pathologies, particularly for recurrent pulmonary infection prophylaxis. Azithromycin has anti-inflammatory, immunomodulatory, and lung remodeling properties in chronic airways disease [45]. Apart from its use in cystic fibrosis, the use in the above indications is off-label. Nevertheless, the literature evidence shows that off-label use is very diffuse [46,47,48].

A proportion of 34.6% of therapy regimens were off-label, with an increasing prevalence in younger patients. Although the evaluation of off-label use criteria may vary depending on the studies, this is a small number compared to other international assessments on this topic, reporting up to 78% of OLDU [22,23,24]. A recent literature review of 47 studies reported an OLDU up to 99.5% among hospitalized pediatric patients whether admitted in the intensive care unit or in the general ward [25]. Notably, our prevalence of OLDU has resulted in lower than the 39.7% reported from Garcia-Lopez et al. in their study involving a total of 85 PPC patients and 1198 prescriptions [21]. The low rate of OLDU reported in this study is probably thanks to the effect of the Italian Law number 648/96 [35]. In addition to this, Italian national authorities have always paid special attention to OLDU. In our case, a double evaluation of OLDU by both the prescribing physician and the authorizing hospital pharmacist made it possible to promptly identify some drugs used outside their registered indications. Of note, off-label drug use was more frequent between patients without medication burden, and a possible explanation for this observation may be that proportionally, the population without medication burden uses fewer drugs, so the percentage of off-label drugs is higher. In fact, the three most used drugs for off-label use are cholecalciferol, melatonin, and lansoprazole, which have a very extensive use in all patients.

Despite our data reporting a lower OLDU compared to other international assessments, they suggest that initiatives to encourage clinical trials for licensing drug use in children are needed to improve the current drug prescribing practices. At the same time, the implementation of legislations about the drug development process and pharmacovigilance could facilitate drug research in the pediatric setting. For instance, the Italian Medicines Agency and the Italian Society of Palliative Care have drawn up two documents that collect the scientifically available evidence to support the off-label use of the most frequently used medicines in pediatric and adult populations [49].

A high variable cost of CMC’s treatment regimens was reported. Remarkably, the highest annual cost was reported in patients with genetic–metabolic conditions at EUR 745,363. This is in contrast with the median daily cost of this diagnostic category of EUR 2.3 (IQR 1.3–9.1). This result can be explained by the high costs of some specific medical regimes in particular for some genetic–metabolic diseases. According to OCSE, the Italian yearly mean pharmaceutical spending is 709 USD/capita, corresponding to 1.9 EUR/capita/day. Remarkably, only 52% (n = 88) of our population have a pharmaceutical spending higher than the average pharmaceutical spending.

However, risdiplam was included in our study but was excluded from the final cost of pharmacological expenses because it was used for compassionate use programs at the time of this study. To date, risdiplam was approved by the Italian Medicines Agency so the costs of the therapies could be different from what is described in this article.

This study has some limitations. First, this is a cross-sectional, non-prospective, monocentric study. Side-effects or adverse reactions related to polypharmacy and OLDU have not been investigated; in addition, an in-depth questionnaire for parents related to the medication burden was not submitted. However, adjunctive and more extended assessments will be the subject of future studies that will provide a more exhaustive characterization of polypharmacy and OLDU among CMC. Furthermore, the possibility to identify and characterize patients with polypharmacy in an effective way can be a starting point of discussion for systematic medication reviews in order to contain and reduce the risk of adverse effects, interaction, and their care burden.

## 5. Conclusions

Polypharmacy and medication burden are frequent among CMC, with significant differences according to primary diagnosis and to care complexities. Since medication burden in children is not yet well defined, particularly in CMCs, it could be critical to estimate adherence to therapy, the risk of administration errors or drug interactions, and the impact on the family’s quality of life.

## Figures and Tables

**Table 1:** Demographic characteristics.

	*N* (%)	Mean (SD)	Median (Min–Max)
Total population	169		
Male	83 (49%)		
Female	86 (51%)		
Age (years)		11.2 (±5.9)	12.5 (0.3–23)
Infant and preschool (0–5 yo)	43 (25.4%)		
School (6–11 yo)	38 (22.5%)		
Adolescent (12–17 yo)	65 (38.5%)		
Adult (≥18 yo)	23 (13.6%)		
Disease history (years)		10.4 (±6.0)	11 (1–23)
DNR ^1^ order	42 (25%)		
Cardiac	2 (1.2%)		
Musculoskeletal	48 (28.4%)		
Neurologic	70 (41.4%)		
Oncological	11 (6.5%)		
Respiratory	11 (6.5%)		
Genetic–metabolic	27 (16.0%)		
Congenital/Perinatal onset	153 (90.5%)		
Polypharmacy ^2^	89 (52.7%)		
Excessive polypharmacy ^3^	33 (19.5%)		
Medication Burden ^4^	75 (44.4%)		
Self-administration	38 (22.5%)		

^1^ DNR: do not resuscitate; ^2^ defined as currency administration of 5 or more drugs per day; ^3^ currency administration of 10 or more drugs per day; ^4^ defined as polypharmacy plus at least 2 drug administration per night.

**Table 2:** The average distribution of the ACCAPED scale score and the distribution of patients by their complexity degree.

	Mean (SD)	*p*-Value	*N *(%)	*p*-Value
Total population	57 (±27)			
Oncological	60 (±9)	0.750		
Non-oncological	57 (±28)			
Infant and preschool (0–5 yo)	61 (±28)	0.061		
School (6–11 yo)	61 (±31)			
Adolescent (12–17 yo)	51 (±22)			
Adult (≥18 yo)	65 (±31)			
Cardiac	51 (±47)	0.009		
Musculoskeletal	46 (±26)			
Neurologic	65 (±28)			
Oncological	60 (±9)			
Respiratory	51 (±28)			
Genetic–metabolic	60 (±26)			
DNR order	72 (±24)	<0.001		
No DNR order	52 (±26)			
Medication burden	68 (±26)	<0.001		
No medication burden	49 (±25)			
Low-complexity-needs patients			27/169 (16%)	<0.001
Middle-complexity-needs patients			38/169 (22.5%)	
High-complexity-needs patients			104/169 (61.5%)	

**Table 3:** Mean number of drugs administered per day, number of drugs as needed, their distribution during the day, and their route of administration (mean ± SD).

	All Patients	Cardiac	Musculoskeletal	Neurologic	Oncological	Respiratory	Genetic-Metabolic
Total drugs	5.7 (±4.0)	8.0 (±1.4)	3.7 (±2.3)	7.5 (±4.3)	4.2 (±4.0)	2.8 (±2.6)	6.5 (±4.0)
Scheduled therapy	5.0 (±3.7)	8.0 (±1.4)	3.1 (±2.2)	6.5 (±3.9)	3.5 (±3.4)	2.3 (±2.3)	5.9 (±3.6)
As needed	0.8 (±1.2)	None	0.6 (±1.3)	1.0 (±1.2)	0.7 (±1.1)	0.6 (±0.9)	0.6 (±1.0)
Daytime drug administrations	5.5 (±4.4)	10.5 (±6.4)	2.9 (±1.8)	7.2 (±4.8)	3.8 (±3.5)	2.6 (±2.7)	6.9 (±4.3)
Nighttime drug administrations	2.7 (±3.0)	3 (±0)	0.9 (±1.6)	4.2 (±3.3)	1.9 (±2.0)	0.9 (±1.4)	3.4 (±2.9)
ATC ^1^ drug classes	3 (±1)	4 (±0)	2.3 (±1.1)	2.9 (±1.3)	2.3 (±1.5)	1.8 (±1.3)	3.0 (±1.2)
Enteral drugs	5.3 (±3.7)	8 (±1.4)	3.4 (±2.2)	6.9 (±3.7)	3.8 (±3.8)	2.3 (±2.2)	6.0 (±3.8)
Parenteral drugs	0.04 (±0.25)	None	None	0.1 (±0.4)	None	None	0.1 (±0.3)
“Other” drugs	0.4 (±0.8)	None	0.2 (±0.6)	0.5 (±0.9)	0.4 (±0.5)	0.6 (±0.8)	0.4 (±0.8)

^1^ ATC = Anatomical Therapeutic Chemical (ATC) classification.

**Table 4:** Prevalence of polypharmacy, self-administration, and medication burden.

	Polypharmacy		Self-Administration		Medication Burden	
	*N* (%)	*p*-Value	*N* (%)	*p*-Value	*N* (%)	*p*-Value
Total population	89/169 (52.7%)		38/169 (22.5%)		75/169 (44.4%)	
Oncological	4/11 (36.4%)	0.264	7/11 (63.6%)	<0.001	5/11 (45.5%)	1.000
Non-oncological	85/158 (53.8%)		31/158 (19.6%)		70/158 (44.3%)	
Infant and preschool (0–5 yo)	21/43 (48.8%)	0.055	3/43 (7%)	<0.001	19/43 (44.2%)	0.146
School (6–11 yo)	20/38 (52.6%)		5/38 (13.2%)		19/38 (50%)	
Adolescent (12–17 yo)	30/65 (46.2%)		24/65 (36.9%)		23/65 (35.4%)	
Adult (≥18 yo)	18/23 (78.3%)		6/23 (26.1%)		14/23 (60.9%)	
Cardiac	2/2 (100%)	<0.001	0/2 (0)	0.001	2/2 (100%)	<0.001
Musculoskeletal	13/48 (27.1%)		19/48 (39.6%)		5/48 (10.4%)	
Neurologic	50/70 (71.4%)		2/70 (2.9%)		44/70 (62.9%)	
Oncological	4/11 (36.4%)		7/11 (63.6%)		5/11 (45.5%)	
Respiratory	3/11 (27.3%)		8/11 (72.7%)		3/11 (27.3%)	
Genetic–metabolic	17/27 (63%)		2/27 (7.4%)		16/27 (59.3%)	
DNR order	32/42 (76.2%)	<0.001	8/42 (19%)	0.38	31/42 (73.8%)	<0.001
No DNR order	57/127 (44.9%)		30/127 (23.6%)		45/127 (35.4%)	

**Table 5:** Annual and daily cost of therapies according to different subpopulations in EUR.

	Median Daily Costs (IQR Range)	Total Daily Costs	Median Annual Costs (IQR Range)	Total Annual Costs	Mann–Whitney Test *p*-Value
Total population	2.2 (0.9–7.1)	3091	792 (332–2630)	1,128,354	
Oncological	3.7 (0.8–16.9)				0.438
Non-oncological	2.2 (0.9–6.9)				
Cardiac	2.9 (2.0–3.9)	6	1066 (722–1409)	2132	
Musculoskeletal	1.5 (0.7–3.0)	114	540 (257–1079)	41,438	
Neurologic	4.5 (1.3–11.8)	745	1650 (489–4333)	271,856	
Oncological	3.7 (0.8–16.9)	168	1333 (234–6337)	61,203	
Respiratory	0.3 (0.1–1.3)	17	102 (41–489)	6362	
Genetic-metabolic	2.3 (1.3–9.1)	2042	821 (457–3635)	745,363	
Infant and preschool (0–5 yo)	1.3 (0.7–5.6)				0.097
School (6–11 yo)	4.0 (1.1–11.0)				
Adolescent (12–17 yo)	1.7 (0.7–4.2)				
Adult (≥18 yo)	6.5 (2.4–13.2)				
DNR order	5.4 (0.5–16.1)				<0.001
No DNR order	1.6 (0.7–5.9)				
Medication burden	6.6 (2.4–13.2)				<0.001
No medication burden	1.1 (0.5–2.5)				

## Data Availability

The datasets used and/or analyzed during the current study are available from the corresponding author on reasonable request due to privacy and ethical restrictions.

## Supplementary Materials

The following supporting information can be downloaded at https://www.mdpi.com/article/10.3390/children11070821/s1, Table S1: Prevalence and details of nervous system drugs.
