# Supplementary material for: Polypharmacy in Children with Medical Complexity: A Cross-Sectional Study in a Pediatric Palliative Care Center
Source: Children (Basel). 2024 Jul 4;11(7):821. doi: 10.3390/children11070821 (PMC11274911; doi:10.3390/children11070821)
Supplement: Supplementary file 1 [file children-11-00821-s001.zip › children-3080026-supplementary.pdf]

Table S1. Nervous system drugs prevalence and details

	**Total nervous system drugs**	**Analgesics**	**Antiepiletics [1]**	**Anti-Parkinsons drugs**	**Psycholeptics**	**Psychoanaleptics**	**Other**
Total population	338/338 (100%)	74/338 (21.9%)	178/338 (52.6%)	4/338 (1.2%)	77/338 (22.8%)	2/338 (0.6%)	3/338 (0.9%)
Cardiac	5/338 (1.6%)	1/74 (1.4%)	2/178 (1.1%)	None	2/77 (2.6%)	None	None
Musculoskeletal	20/338 (6.0%)	6/74 (8.2%)	5/178 (2.8%)	None	7/77 (9.1%)	1/2 (50%)	1/3 (33.3%)
Neurologic	232/338 (68.6%)	36/74 (48.6%)	134/178 (75.3%)	3/4 (75%)	56/77 (72.7%)	1/2 (50%)	2/3 (66.7%)
Oncological	23/338 (6.8%)	14/74 (18.9%)	7/178 (3.9%)	None	2/77 (2.6%)	None	None
Respiratory	3/338 (1%)	None	1/178 (0.6%)	None	2/77 (2.6%)	None	None
Genetic-metabolic	54/338 (16%)	17/74 (22.9%)	29/178 (16.3%)	1/4 (25%)	8/77 (10.4%)	None	None

[1] Including gabapentinoids (n=18, 5%)
